# What genome-wide association studies reveal about the association between intelligence and physical health, illness, and mortality

**DOI:** 10.1016/j.copsyc.2018.07.005

**Published:** 2019-06

**Authors:** Ian J Deary, Sarah E Harris, W David Hill

**Affiliations:** 1 Centre for Cognitive Ageing and Cognitive Epidemiology, Department of Psychology, University of Edinburgh, 7 George Square, Edinburgh EH8 9JZ, United Kingdom; 2 Medical Genetics Section, Centre for Genomic & Experimental Medicine, MRC Institute of Genetics & Molecular Medicine, University of Edinburgh, Western General Hospital, Edinburgh EH4 2XU, United Kingdom

## Abstract

The associations between higher intelligence test scores from early life and later good health, fewer illnesses, and longer life are recent discoveries. Researchers are mapping the extent of these associations and trying to understanding them. Part of the intelligence-health association has genetic origins. Recent advances in molecular genetic technology and statistical analyses have revealed that: intelligence and many health outcomes are highly polygenic; and that modest but widespread genetic correlations exist between intelligence and health, illness and mortality. Causal accounts of intelligence-health associations are still poorly understood. The contribution of education and socio-economic status — both of which are partly genetic in origin — to the intelligence-health associations are being explored.

**Current Opinion in Psychology** 2019, **27**:6–12

This review comes from a themed issue on **Genetics**

Edited by **Brian B Boutwell** and **Michael A White**

For a complete overview see the Issue and the Editorial

Available online 23rd July 2018

**https://doi.org/10.1016/j.copsyc.2018.07.005**

2352-250X/© 2018 The Authors. Published by Elsevier Ltd. This is an open access article under the CC BY license (http://creativecommons.org/licenses/by/4.0/).

## Intelligence, and health and death

Until recently, an article on DNA-variant commonalities between intelligence and health would have been science fiction. Thirty years ago, we did not know that intelligence test scores were a predictor of mortality. Fifteen years ago, there were no genome-wide association studies. It was less than five years ago that the first molecular genetic correlations were performed between intelligence and health outcomes. These former blanks have been filled in; however, the fast progress and accumulation of findings in the field of genetic cognitive epidemiology have raised more questions. Individual differences in intelligence, as tested by psychometric tests, are quite stable from later childhood through adulthood to older age [1,2]. The diverse cognitive test scores that are used to test mental capabilities form a multi-level hierarchy [1, 2, 3]; about 40% or more of the overall variance is captured by a general cognitive factor with which all tests are correlated, and smaller amounts of variance are found in more specific cognitive domains (reasoning, memory, speed, verbal, and so forth). Twin, family and adoption studies indicated that there was moderate to high heritability of general cognitive ability in adulthood (from about 50–70%), with a lower heritability in childhood [4]. It has long been known that intelligence is a predictor of educational attainments and occupational position and success [1].

Relatively recently, the ‘ultimate validity’ of intelligence test scores was discovered, that is, that higher intelligence significantly predicts later death. First, an Australian Vietnam Veterans study found that higher young-adult intelligence predicted lower risk of accidental deaths up to early middle age [5]. Then, a population-representative Scottish study found that intelligence test scores at age 11 years predicted deaths from all causes up to older age (the mid-70 s) [6]. The association between intelligence test scores from early life and mortality from all causes has been widely replicated [7, 8, 9]. Intelligence from childhood and adulthood is associated with most of the major causes of death with the exception of non-smoking-related cancers [10^••^,^11^]. Broadly speaking, a one-standard-deviation advantage in intelligence in youth lowers the risk of mortality by 20–25% or more up to older age; the effect sizes are hardly attenuated at all by adjusting for childhood socio-economic status, though are partly attenuated after adjusting for education and adult socio-economic status, which are possible mediators of the association [6,7,8,9,10^••^,^11^].

In addition to mortality, intelligence test scores are associated with lower risk of many morbidities, such as cardiovascular disease, cerebrovascular disease, hypertension, cancers such as lung cancer, stroke, and many others, as obtained by self-report and objective assessment [12, 13, 14]. Higher intelligence in youth is associated at age 24 with fewer hospital admissions, lower general medical practitioner costs, lower hospital costs, and less use of medical services, and intelligence appeared to account for the associations between education and such health outcomes [15,16]. Higher intelligence is related to a higher likelihood of engaging in healthier behaviours, such as not smoking, quitting smoking, not binge drinking, having a more normal body mass index and avoiding obesity, taking more exercise, and eating a healthier diet [16, 17, 18].

The flood of intelligence versus mortality/illness/health-behaviours findings was captured by the term ‘cognitive epidemiology’ [19]. From early on until now, there have been speculations about the possible causes of these associations [6,10^••^,^14^,^20^]. Briefly, there is acknowledgement that the causes of the associations are probably multiple, such as there being a constitutional (perhaps partly genetic) association between intelligence and health, and/or that intelligence’s influence might act via more education, higher health literacy, and more affluent social class. Here, we examine evidence for possible genetic links between intelligence and health.

## Genetic contributions to health, and to intelligence

There are at least three reasons to conduct genetic studies of phenotypes. First one wants to understand the genetic architecture of a phenotype, that is, what is the nature of the genetic variants that contribute to variation in the phenotype. For example, a single mutation might have a large effect, as is the case in Mendelian diseases. By contrast, continuous traits might be more likely to be polygenic; that is, to have some of their variance caused by small contributions from many genetic variants. Second, having discovered the genetic architecture, one is interested in the specific genes in which variants have causal effects, that is, one wants to understand the molecular genetic mechanisms of variation. Third, knowing that there is some genetic contribution to a phenotype, one can ask how good a predictor the genotypic information is; that is, how well can one predict some variation in a phenotype from only genotypic information? Much recent progress has been made along these lines for illnesses and for intelligence.

Before the mid-2000s, genetic studies were done by three main methods. First, pedigree-based (twins, adoptees, and families) studies of relatives’ phenotypic associations were used to estimate the heritability of phenotypes, and genetic correlations among them. Limitations of pedigree methods include the fact that several assumptions must be made in doing the modelling, and that one does not learn about the specific genes involved. Second, candidate gene studies tested hypotheses concerning whether certain genetic variants were associated with phenotypic differences. For example, the possession of the e4 allele of the gene for Apolipoprotein E (*APOE*) is associated with an increased risk of developing Alzheimer’s disease. Limitations of the candidate gene method include the fact that most candidate gene findings are not replicated (*APOE* e4 possession is an exception to this), and that it is difficult to choose a candidate genetic variant from the millions that are known. Third, genetic linkage analysis was used to track genetic markers in families where specific phenotypes were common, to identify regions of the genome that segregate with the phenotype. The main limitations of this method are that large families are required and it identifies relatively large regions of the genome, rather than specific genetic variants or genes.

This changed with the advent and rise of genome-wide association studies (GWASs) [21] (See Box 1). Sample sizes for GWASs often began with a few thousand, but, as the polygenic architecture of many traits became clear — , that is, the associations between individual genetic variants and phenotypes typically had very small effect sizes — it was necessary to form consortia so that the Ns of studies rose to the tens and then hundreds of thousands. Some GWAS consortia are now approaching and passing one million participants.

Box 1 **Methods for investigating shared genetic aetiology between intelligence and physical health, illness and mortality**

1. Genome-wide association study (GWAS)

GWAS is used to identify genetic variants associated with phenotypes. For diseases, large numbers of cases and controls are genotyped using testing arrays, most commonly from the companies Illumina or Affymetrix. The arrays contain up to 1 million genetic variants spread throughout the genome. For quantitative traits, large numbers of individuals on whom the trait is measured are genotyped. Using reference datasets, for example, Hap Map, 1000 Genomes and the haplotype reference consortium (HRC), several million genetic variants are then imputed to give greater genomic coverage and to harmonize datasets genotyped using different genetic testing arrays, containing different variants. Logistic (for case control) or linear (for quantitative traits) regressions are then performed between each genetic variant and the phenotype. As millions of regressions are performed for each phenotype a *P*-value of <5 × 10^−8^ is usually considered genome-wide significant. As GWASs do not analyse every variant in the genome they do not usually identify causative variants, but rather indicate regions of the genome that are implicated in a particular phenotype. If the same regions of the genome are identified in GWASs of multiple phenotypes this may indicate that the phenotypes share genetic aetiology.

2. Genome-wide complex trait analysis (GCTA)**-**genomic-relatedness-based restricted maximum-likelihood single component (GREML)

GREML [26] is used to estimate the proportion of variance in a phenotype that is due to the linkage disequilibrium between genotyped variants and unknown causal variants. It gives a lower-boundary estimate of the heritability of a phenotype as it does not include variance accounted for by genetic variants that are not well tagged by variants on the array, for example, rare variants.

3. Bivariate GCTA

Bivariate GCTA [46] is an extension of GCTA-GREML that allows the genetic correlation between two phenotypes to be determined. Genetic correlation describes the proportion of the variance that two phenotypes share that is due to genetic causes. High genetic correlation between two phenotypes indicates that the phenotypes share genetic aetiology. This method requires the actual genotyping data from the sample and for the sample to have been measured on the two phenotypes in question.

4. Linkage disequilibrium (LD) regression

LD regression [30,31] is a method that can be used to determine the genetic correlation between two phenotypes, using only summary statistics from GWASs. LD regression estimates the genetic effect on a trait by measuring the extent to which the observed effect sizes from a GWAS can be explained by LD. The covariance between the genetic effects in two phenotypes can be indexed in a similar way; normalizing this genetic covariance by the heritability of the trait will estimate the genetic correlation between the two traits. This method does not require genotyping data, and can produce genetic correlations for two phenotypes that were GWAS-ed on different samples. For example LD regression is used to compute the genetic correlation between intelligence and longevity by using summary statistics from a GWAS of mortality and a GWAS of intelligence, both conducted on independent samples.

5. Polygenic scores (PGS)

PGS analysis uses summary GWAS data for a given phenotype to test whether polygenic liability to that phenotype is associated with the same or different phenotype measured in an independent sample. It allows the amount of variance in one phenotype attributed to the polygenic score for the same or a second phenotype to be calculated. A PGS for a particular phenotype can be calculated for each individual in a sample, by summing the known effect size of each individual SNP (obtained from a GWAS of that phenotype) multiplied by the number of reference alleles present for that SNP in a particular individual. PGSs can be calculated using PRSice [47]. For example, the polygenic score for risk of coronary artery disease is associated with cognitive ability in older adults [48].

6. Mendelian Randomization (MR)

The methods described above will indicate whether or not two phenotypes share genetic aetiology, but do not reveal the direction of causation. Once shared genetic aetiology between two phenotypes is established, MR methods can be used to investigate whether one phenotype directly influences the other phenotype, or whether genetic variants independently affect both phenotypes. Bi-directional MR allows each phenotype to be used as the exposure or the outcome in turn, potentially providing support for the direction of effect. In MR, genetic variants (often variants that are genome-wide significantly associated with the relevant phenotype) are used as instrumental variables (IV) for the exposure. Unlike the exposure itself, these genetic variants should be largely independent of confounding factors and reverse causation. The IV is used to estimate if the exposure causally influences the outcome. There are three basic assumptions of MR: Firstly, the genetic variants are associated with the exposure; secondly, the genetic variants are only associated with the outcome of interest via their effect on the exposure; and finally, the genetic variants are independent of confounders of both the exposure and the outcome. Biological pleiotropy, whereby a genetic variant independently influences multiple traits, may violate the second assumption. The more SNPs that make up the IV the more likely that biological pleiotropy will be present. Methods have now been developed that test and correct for biological pleiotropy [49]. Two-sample MR allows the exposure and the outcome to be measured in different samples and therefore the effect of the IV on the exposure and outcome can be obtained from GWAS summary data [50].

Alt-text: Box 1

The typical finding — there are exceptions — in health and cognitive GWASs is that many genetic variants of small effect contribute to phenotypic variation. In 2017, a survey of the first ten years of GWASs’ discoveries enumerated the SNPs that were associated with, for example, Crohn’s disease, diabetes, blood lipid levels, heart function, height, bone density, red blood cell traits, metabolic traits, blood platelets, breast cancer, rheumatoid arthritis, blood metabolites, menarche, Alzheimer disease, kidney function, lung function, and education [21]. Often, the numbers of genetic loci in which significant SNP associations are found runs to dozens or even hundreds for a single phenotype.

**Figure 1:**
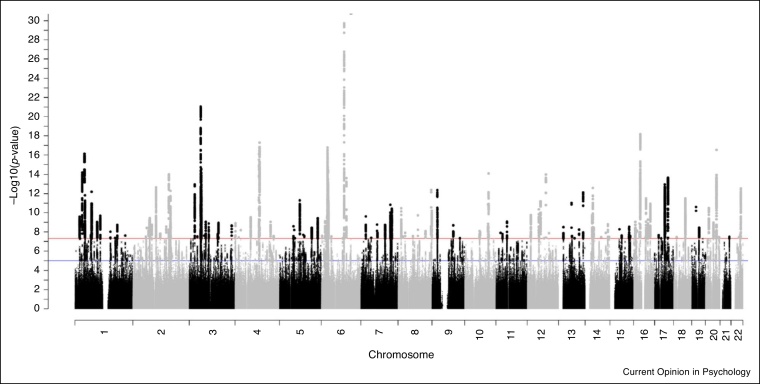
This is a ‘Manhattan plot’, taken from Davies *et al.*’s genome-wide association study of 300,486 participants on general cognitive function [24^•^]. Chromosome number is shown on the *X*-axis, with each dot representing one of the more than 12 million imputed single nucleotide polymorphisms. The red line represents genome-wide significance, that is, *P* < 5 × 10^−8^.

In 2011 the first apparently-decently-sized GWAS of intelligence appeared (N approximately 3500), and found no significant SNPs [22]. By the time the sample size was about 100 times greater, the number of independent genomic regions that were associated with intelligence was about or greater than 150 [23^•^,24^•^,25^••^]. Figure 1 shows results from a recent GWAS of intelligence. Many of these SNPs are located in regions of the genome that have previously been associated with physical and mental illnesses. Therefore, we now know many actual DNA variants that have significant associations with intelligence tests’ scores; there are probably thousands in total. Although it found no significant SNPs, the 2011 paper [22] did make a difference; it was the first study to estimate the heritability of intelligence from DNA data alone and in unrelated subjects. This used a then-new method—called GREML, and run in the GCTA framework [26] — which examined people’s overall genetic similarity — based on common SNPs — with their phenotypic similarity (See Box 1). The common-SNP-based heritability of intelligence is estimated to be about 25% [24^•^]. It is typical for this common-SNP-based heritability to be about half of that estimated from twin studies [27^•^]. It is thought that this ‘missing heritability’ is because there are types of genetic variants other than causal variants that are in linkage disequilibrium with common SNPs that contribute to heritability. Some new techniques are helping to find these and close the gap between twin-based and SNP-based heritability [28].

## Genetics and the intelligence-health relationship

Three things are clear. First, higher intelligence in early life is a significant predictor of better health behaviours, fewer and later illnesses, and longer life. Second, many of the relevant health and illness outcomes, as well as health behaviours, have many SNPs associated with them, and have a detectable level of common-SNP-based heritability. Third: the same goes, genetically, for intelligence. Relatively new methods — bivariate extension of GREML run on GCTA [29], and LD regression [30,31] (see Box 1) — have allowed estimates of the genetic correlations between phenotypes. That is, we can test the extent to which the polygenic signature obtained by using the summary results from GWAS contributes to any two phenotypes, including between intelligence and health. Polygenic signatures for many diseases were soon shown to be associated with intelligence [32]. Twin studies had suggested that part of the intelligence-mortality association might be genetic in origin, though there was disagreement about how much genetics contributed [33,34]. However, more recent studies have used genomic data.

The list of significant molecular genetic correlations between intelligence and physical health variables is now long [23^•^,24^•^,25^••^,^32^]. Table 1 gives some examples. With regard to mortality, longevity has been used; parental age at death has also been used, as a proxy, because most relevant studies have not carried on long enough for many participants to have died. There is a positive correlation of 0.36 between intelligence and parental age at death. There are inverse genetic correlations between intelligence and both heart disease and hypertension, with effect sizes between −0.1 and −0.2. There is a small (<0.1) association with cholesterol, with higher ‘good’ cholesterol going with higher intelligence and the reverse for the ‘bad’ cholesterol. There is a moderate-sized inverse genetic association between intelligence and Alzheimer’s disease. There is a positive genetic association, of 0.27, with intracranial volume, which is an indication of maximal brain volume in the life course. There are significant positive genetic correlations between intelligence and birth weight, lung function, happiness, and short-sightedness. There are significant negative genetic correlations between intelligence and body mass index, poor self-rated health, lung cancer, osteoarthritis, insomnia, smoking, waist-hip ratio, and long-sightedness. It must be stressed that these correlations are based on GWASs conducted on different samples; that is the people on whom intelligence was measured were not the people on whom the health-based phenotype was assessed. Associations are interesting, but they do not explain why the correlations exist, or the direction of causation, which require further study and more new GWAS-based methods.

**Table 1:** Genetic correlations (rg, with *P* values) between intelligence and health outcomes from Hill 2018 [23^•^], Davies 2018 [24^•^], and Savage [25^••^].

Phenotype category	Phenotype	Hill, 2018 [23^•^]		Davies, 2018 [24^•^]		Savage, 2018 [25^••^]	
		rg	*P*	rg	*P*	rg	*P*
Vascular-metabolic	Angina			−0.18	7.58 × 10^−9^		
	Coronary artery disease	−0.17	1.03 × 10^−6^	−0.11	0.0024	−0.19	1.23 × 10^−7^
	HDL cholesterol			0.092	8.10 × 10^−6^		
	Heart attack			−0.17	4.74 × 10^−7^		
	Hypertension			−0.15	2.44 × 10^−14^		
	LDL cholesterol			−0.055	0.019		
	Triglycerides			−0.052	0.027		
	Type 2 diabetes	−0.09	0.0077	−0.073	0.043	−0.042	0.33

Brain measures	Infant head circumference					0.28	7.18 × 10^−5^
	Intracranial volume			0.27	1.56 × 10^−5^	0.25	6.56 × 10^−9^

Physical and physiological	Age at Menopause			0.13	1.10 × 10^−5^		
	Alzheimer's disease	−0.38	0.00010	−0.37	2.78 × 10^−5^	−0.26	3.95 × 10^−5^
	Birth length					0.16	3.88 × 10^−3^
	Birth weight			0.11	2.09 × 10^−6^	0.10	0.12
	BMI	−0.16	1.38 × 10^−16^	−0.13	8.38 × 10^−12^	−0.12	1.64 × 10^−6^
	Chronotype	−0.15	1.45 × 10^−8^				
	Fathers age at death			0.37	4.75 × 10^−33^		
	FEV1	0.10	0.00037	0.19	1.33 × 10^−19^		
	Hand grip strength (right)			0.086	4.08 × 10^−5^		
	Happiness			0.086	9.2 × 10^−3^		
	Head circumference	0.31	1.33 × 10^−8^				
	Health satisfaction			−0.26	3.43 × 10^−21^		
	Height	0.12	1.13 × 10^−14^			0.082	4.96 × 10^−4^
	Longevity			0.17	0.0036	0.43	4.91 × 10^−8^
	Lung cancer			−0.26	4.73 × 10^−9^		
	Mothers age at death			0.48	5.82 × 10^−30^		
	Obesity	−0.18	9.3 × 10^−14^				
	Osteoarthritis			−0.24	3.78 × 10^−11^		
	Overall poor health rating			−0.39	7.69 × 10^−105^		
	Parents age at death	0.37	0.0094	0.36	3.5 × 10^−10^		
	Self-rated health	0.46	1.37 × 10^−83^				
	Sleeplessness/insomnia			−0.12	7.27 × 10^−8^		
	Smoking status	−0.27	2.9 × 10^−14^	−0.20	5.61 × 10^−8^	−0.15	2.61 × 10^−3^
	Tiredness	−0.18	8.28 × 10^−9^				
	Waist circumference					−0.10	9.69 × 10^−5^
	Waist-hip ratio			−0.17	4.02 × 10^−14^	−0.17	8.6 × 10^−10^
	Wears glasses or contact lenses			0.28	2.12 × 10^−13^		
	Longsighted-ness			−0.21	2.04 × 10^−5^		
	Shortsighted-ness			0.32	1.92 × 10^−24^		

*Note*: Samples contributing to the three papers are not independent. Variables with no significant genetic correlation with intelligence in any of the three studies were not included. *P* values shown are nominal; some do not survive Bonferroni correction or correction for false discovery rate, as indicated in the three papers.

## Understanding the intelligence versus physical health association, including the part played by genetics

As described above, genetic correlations have been identified between intelligence and many diseases, and physical health traits; moreover, polygenic scores for diseases and health traits predict intelligence. However, it is not clear if these findings are due to: (1) genetic variants influencing health traits/diseases, and then those health traits/diseases influencing intelligence; (2) genetic variants influencing intelligence, and then intelligence influencing health traits/diseases; or (3) genetic variants influencing general bodily system integrity [20] that influences both intelligence and health traits/diseases. (1) and (2) may be due to mediated pleiotropy which can be tested for using a relatively new technique called Mendelian Randomization (MR) (see Box 1).

Using a bi-directional two-sample MR approach we identified no causal association between intelligence or educational attainment (a proxy measure of intelligence), and the physical health traits of body mass index (BMI), systolic blood pressure, height, coronary artery disease and type 2 diabetes [35], using data from the UK Biobank (N approximately 110,000) and large GWAS consortia. However, a larger, more-recent study found MR-based evidence for potentially causal genetic effects of intelligence on larger intracranial volume, lower risk of Alzheimer’s disease, lower body mass index, and greater likelihood of quitting smoking [25^••^]. A MR study investigating the effect of education on obesity in about 2000 Finns concluded that education could be a protective factor against obesity, as measured using BMI [36]. Another study using education data from the SSGAC consortium and coronary heart disease data from CARDIoGRAMplusC4D (total sample size = 543,733) found that higher education was causally associated with reduced risk of coronary heart disease, lower likelihood of smoking, lower BMI and a more favourable blood lipid profile [37]. Sensitivity tests indicated that the results were unlikely to be driven by biological pleiotropy. A two-step MR study investigated the influence of vitamin B12 intake during pregnancy on cord blood DNA methylation and whether there is a causal influence on offspring’s cognition in the Avon Longitudinal Study of Parents and Children (ALSPAC) [38]. A small causal effect of vitamin B12-responsive DNA methylation changes on children’s cognition was identified. MR analysis has suggested that genetically-predicted intelligence and education both had associations with Alzheimer’s disease [39].

Another part of understanding the genetic contribution to intelligence-health correlations concerns other predictors of health inequalities, and intelligence’s correlations with them. Intelligence, we saw earlier, is related to education and socio-economic status (SES), and those were known to be related to health inequalities before intelligence was known to have health associations. Although education and SES are principally thought of as social-environmental variables, both have been found to be partly heritable, by both twin-based and molecular genetic studies, both have high genetic correlations with intelligence, Mendelian Randomisation results show bidirectional genetic effects between intelligence and education, and both have genetic correlations with health outcomes [25^••^,^40^,^41^,^42^,^43^,^44^,^45^].

## Conclusion

Intelligence has predictive power for many health outcomes. Part of that association is genetic. The genes involved, and the causal pathways of the associations are being explored.

## Funding

IJD and SEH are supported by the University of Edinburgh Centre for Cognitive Ageing and Cognitive Epidemiology which is funded by the UK Medical Research Council and Biotechnology and Biological Sciences Research Council (Grant no. MR/K026992/1). WDH is supported by Age UK (Disconnected Mind programme).

## Conflict of interest statement

Nothing declared.

## References and recommended reading

Papers of particular interest, published within the period of review, have been highlighted as:

• of special interest
•• of outstanding interest

## References

[1] Deary I.J. (2012). Intelligence. Ann Rev Psychol.

[2] Deary I.J. (2013). Intelligence. Curr Biol.

[3] Carroll J.B. (1993). Human Cognitive Abilities: A Survey of Factor-Analytic Studies.

[4] Haworth C.M.A., Wright M.J., Luciano M., Martin N.G., de Geus E.J.C., van Beijsterveldt C.E.M., Bartels M., Posthuma D., Boomsma D.I., Davis O.S.P. (2010). The heritability of general cognitive ability increases linearly from childhood to young adulthood. Mol Psychiatry.

[5] O’Toole B.I., Stankov L. (1992). Ultimate validity of psychological tests. Pers Individ Dif.

[6] Whalley L.J., Deary I.J. (2001). Longitudinal cohort study of childhood IQ and survival up to age 76. Br Med J.

[7] Cukic I., Brett C.E., Calvin C.M., Batty G.D., Deary I.J. (2017). Childhood IQ and survival to 79: follow-up of 94% of the scottish mental survey 1947. Intelligence.

[8] Calvin C.M., Deary I.J., Fenton C., Roberts B., Der G., Leckenby N., Batty G.D. (2011). Intelligence in youth and all-cause mortality: systematic review with meta-analysis. Int J Epidemiol.

[9] Bratsberg B., Rogeberg O. (2017). Childhood socioeconomic status does not explain the IQ-mortality gradient. Intelligence.

[10••] Calvin C., Batty G.D., Der G., Brett C.E., Pattie A., Cukic I., Deary I.J. (2017). Childhood intelligence in relation to major causes of death in 68 year follow-up: prospective population study. Br Med J.

[11] Batty G.D., Deary I.J., Zaninotto P. (2016). Association of cognitive function with cause-specific mortality in middle and older age: follow-up of participants in the English Longitudinal Study of Ageing. Am J Epidemiol.

[12] Wraw C., Deary I.J., Gale C.R., Der G. (2015). Intelligence in youth and health at age 50. Intelligence.

[13] Backhouse E.V., McHutchison C.A., Cvoro V., Shenkin S.D., Wardlaw J.M. (2017). Early life risk factors for cerebrovascular disease: a systematic review and meta-analysis. Neurology.

[14] Deary I.J., Weiss A., Batty G.D. (2010). Intelligence and personality as predictors of illness and death: how researchers in differential psychology and chronic disease epidemiology are collaborating to understand and address health inequalities. Psychol Sci Public Interest.

[15] Kraft M., Arts K., Traag T., Otten F., Bosma H. (2018). The contribution of intellectual abilities to young adult's educational differences in health care use: a prospective cohort study. Intelligence.

[16] Caspi A., Houts R.M., Belsky D.W., Harrington H., Hogan S., Ramrakha S., Poulton R., Moffitt T. (2016). Childhood forecasting of a small segment of the population with large economic burden. Nat Hum Behav.

[17] Wraw C., Der G., Gale C.R., Deary I.J. (2018). Intelligence in youth and health behaviours in middle age. Intelligence.

[18] Batty G.D., Deary I.J., Schoon I., Gale C.R. (2007). Childhood mental ability in relation to food intake and physical activity in adulthood: the 1970 British Cohort Study. Pediatrics.

[19] Deary I.J. (2010). Cognitive epidemiology: its rise, its current issues, and its challenges. Pers Individ Diff.

[20] Deary I.J. (2012). Looking for ‘system integrity' in cognitive epidemiology. Gerontology.

[21] Visscher P.M., Wray N.R., Zhang Q., Sklar P., McCarthy M.I., Brown M.A., Yang J. (2017). 10 years of GWAS discovery: biology, function, and translation. Am J Hum Genet.

[22] Davies G., Tenesa A., Payton A., Yang J., Harris S.E., Liewald D., Ke X., Le Hellard S., Chistoforou A., Luciano M. (2011). Genome-wide association studies establish that human intelligence is highly heritable and polygenic. Mol Psychiatry.

[23•] Hill W.D., Marioni R., Maghzian O., Ritchie S., Hagenaars S., McIntosh A., Gale C., Davies G., Deary I.J. (2018). A combined analysis of genetically correlated traits identifies 187 loci and a role for neurogenesis and myelination in intelligence. Mol Psychiatry.

[24•] Davies G., Lam M., Harris S.E., Trampush J.W., Luciano M., Hill W.D., Hagenaars S.P., Ritchie S.J., Marioni R.E., Fawns-Ritchie C. (2018). Study of 300,486 individuals identifies 148 independent genetic loci influencing general cognitive function. Nat Commun.

[25••] Savage J.E., Jansen P.R., Stringer S., Wantanabe K., Bryois J., de Leeuw C.A., Nagel M., Awasthi S., Barr P., Coleman J.R.I. (2018). Genome-wide association meta-analysis in 269,867 individuals identifies new genetic and functional links to intelligence. Nat Genet.

[26] Yang J., Benyamin B., McEvoy B.P., Gordon S., Henders A.K., Nyholt D.R., Madden P.A., Heath A.C., Martin N.G., Montgomery G.W. (2010). Common SNPs explain a large proportion of the heritability for human height. Nat Genet.

[27•] Plomin R., von Stumm S. (2018). The new genetics of intelligence. Nat Rev Genet.

[28] Hill W.D., Arslan R.C., Xia C., Luciano M., Amador C., Navarro P., Hayward C., Nagy R., Porteous D.J., McIntosh A.M. (2018). Genomic analysis of family data reveals additional genetic effects on intelligence and personality. Mol Psychiatry.

[29] Deary I.J., Davies Y.J., Harris G., Tenesa S.E., Liewald A., Luciano D., Lopez L.M., Gow A.J., Caorley J. (2012). Genetic contributions to stability and change in intelligence from childhood to old age. Nature.

[30] Bulik-Sullivan B.K., Loh P.-R., Finucane H.K., Ripke S., Yang J., Patterson N., Daly M.J., Price A.L., Neale B.M. (2015). Consortium SWGotPG: Ld score regression distinguishes confounding from polygenicity in genome-wide association studies. Nat Genet.

[31] Bulik-Sullivan B., Finucane H.K., Anttila V., Gusev A., Day F.R., Loh P.-R., ReproGen Consortium, Psychiatric Genomics Consortium, Genetic Consortium for Anorexia Nervosa of the Wellcome Trust Case Control Consortium, Duncan L., Perry J.R.B. (2015). An atlas of genetic correlations across human diseases and traits. Nat Genet.

[32] Hagenaars S.P., Harris S.E., Davies G., Hill W.D., Liewald D.C.M., Ritchie S.J., Marioni R.E., Fawns-Ritchie C., Cullen B., Malik R. (2016). Shared genetic aetiology between cognitive functions and physical and mental health in UK Biobank (*N* = 112151) and 24 GWAS consortia. Mol Psychiatry.

[33] Arden R., Luciano M., Deary I.J., Reynolds C.A., Pedersen N.L., Plassman B., McGue M., Christensen K., Visscher P.M. (2016). The association between intelligence and lifespan is mostly genetic. Int J Epidemiol.

[34] Christensen G.T., Osler M., Madsen M., McGue M., Mortensen E.L., Christensen K. (2017). The influence of familial factors on the intelligence-mortality association — a twin approach. Intelligence.

[35] Hagenaars S.P., Gale C.R., Deary I.J., Harris S.E. (2017). Cognitive ability and physical health: a Mendelian randomization study. Sci Rep.

[36] Böckerman P., Viinikainen J., Pulkki-Råback L., Hakulinen C., Pitkänen C., Lehtimäki T., Pekhonen J., Raitakari O.T. (2017). Does higher education protect against obesity? Evidence using Mendelian randomization. Prev Med.

[37] Tillmann T., Vaucher J., Okbay A., Pikhart H., Peasey A., Kubinova R., Pajak A., Tamosiiunas A., Malyutina S., Hartwig F.P. (2017). Education and coronary heart disease: Mendelian Randomisation study. Br Med J.

[38] Caramaschi D., Sharp G.C., Nohr E.A., Berryman K., Lewis S.J., Davey Smith G., Relton C.L. (2017). Exploring a causal role of DNA methylation in the relationship between maternal vitamin B12 during pregnancy and child's IQ at age 8, cognitive performance and educational attainment: a two-step Mendelian randomization study. Hum Mol Gen.

[39] Larsson S.C., Traylor M., Malik R., Dichgans M., Burgess S., Markus H.S. (2017). Modifiable pathways in Alzheimer's disease: mendelian randomisation analysis. Br Med J.

[40] Calvin C.M., Deary I.J., Webbink D., Smith P., Fernandes C., Lee S.H., Luciano M., Visscher P.M. (2012). Multivariate genetic analyses of cognition and education from two population samples of 174,000 and 166,000 school children. Behav Genet.

[41] Okbay A., Beauchamp J.P., Fontana M.A., Lee J.J., Pers T.H., Rietveld C.A., Turley P., Chen G.B., Emilsson V., Meddens S.F. (2016). Genome-wide association study identifies 74 loci associated with educational attainment. Nature.

[42] Hill W.D., Hagenaars S.P., Marioni R.E., Harris S.E., Liewald D.C.M., Davies G., Okbay A., McIntosh A.M., Gale C.R., Deary I.J. (2016). Molecular genetic contributions to social deprivation and household income in UK Biobank. Curr Biol.

[43] Belsky D.W., Moffitt T.E., Corcoran D.L., Domingue B., Harrington H., Hogan S., Houts R., Ramrakja S., Sugden K., Williams B.S. (2016). The genetics of success: how single-nucleotide polymorphisms associated with educational attainment relate to life course development. Psychol Sci.

[44] Krapohl E., Plomin R. (2016). Genetic link between family socioeconomic status and children's educational achievement estimated from genome-wide SNPs. Mol Psychiatry.

[45] Marioni R.E., Ritchie S.J., Joshi P.K., Hagenaars S.P., Okbay A., Fischer K., Adams M.J., Hill W.D., Davies G., Nagy R. (2016). Genetic variants linked to education predict longevity. Proc Natl Acad Sci U S A.

[46] Lee S.H., Yang J., Goddard M.E., Visscher P.M., Wray N.R. (2012). Estimation of pleiotropy between complex diseases using single-nucleotide polymorphism-derived genomic relationships and restricted maximum likelihood. Bioinformatics.

[47] Euesden J., Lewis C.M., O’Reilly P.F. (2015). PRSice: polygenic risk score software. Bioinformatics.

[48] Hagenaars S.P., Harris S.E., Clarke T.K., Hall L., Luciano M., Fernandez-Pujals A.M., Davies G., Hayward C., Generation Scotland, Starr J.M. (2016). Polygenic risk for coronary artery disease is associated with cognitive ability in older adults. Int J Epidemiol.

[49] Bowden J., Davey Smith G., Burgess S. (2015). Mendelian randomization with invalid instruments: effect estimation and bias detection through Egger regression. Int J Epidemiol.

[50] Burgess S., Scott R.A., Timpson N.J., Davey Smith G., Thompson S.G. (2015). EPIC – InterAct Consortium. Using published data in Mendelian randomization: a blueprint for efficient identification of causal risk factors. Eur J Epidemiol.

